# The 3′ UTR Variants in the *GRP78* Are Not Associated with Overall Survival in Resectable Hepatocellular Carcinoma

**DOI:** 10.1371/journal.pone.0017783

**Published:** 2011-03-22

**Authors:** Xiao Zhu, Fang Wang, Marie C. M. Lin, Linwei Tian, Wenguo Fan, Samuel S. Ng, Minjuan Liu, Jianqing Huang, Zhenhua Xu, Dongpei Li, Hsiangfu Kung

**Affiliations:** 1 Cancer Institute and Affiliated Tumor Hospital, Guangzhou Medical College, Guangzhou, China; 2 State Key Laboratory of Oncology in South China, Cancer Center, Sun Yat-sen University, Guangzhou, China; 3 Department of Surgery, Prince of Wales Hospital, The Chinese University of Hong Kong, Hong Kong, China; 4 Department of Epidemiology, School of Public Health and Primary Care, The Chinese University of Hong Kong, Hong Kong, China; 5 Zhongshan School of Medicine, Sun Yat-sen University, Guangzhou, China; 6 Department of Biology, The University of Hong Kong, Hong Kong, People's Republic of China; 7 Li Ka Shing Institute of Medical Sciences, The Chinese University of Hong Kong, Hong Kong, China; Ohio State University Medical Center, United States of America

## Abstract

**Background:**

Elevated glucose-regulated protein 78 (GRP78) levels in tissues have been known to be related with poor prognosis in hepatocellular carcinoma (HCC) patients. Though the variants in the 3′ untranslated region (UTR) of *GRP78* gene were not associated with HCC risk, we wonder whether these polymorphisms affect survival of HCC patients.

**Methodology/Principal Findings:**

Blood samples of HCC patients were maintained in our specimen bank between 1996 to 2003. DNA from 576 unrelated and resectable patients with HCC was typed for rs16927997 (T>C), rs1140763 (T>C) and rs12009 (T>C) by TaqMan assays. The Kaplan-Meier method and log-rank test were used to estimate overall survival. Linkage disequilibrium (LD) analysis identified a total of 3 haplotypes and 6 diplotypes in this region. The distribution of haplotype was not related to the clinical characteristics. Univariate analysis showed that the allele, genotype, haplotype and diplotype did not effect the survival. None of the clinical features show a significant association (*P*_correced_>0.05) with overall patient outcome in multiple comparisons.

**Conclusions/Significance:**

There is no noteworthy influence of 3′ UTR variants in the *GRP78* on prognosis of resectable HCC in the Chinese population.

## Introduction

Incidence is increasing and hepatocellular carcinoma (HCC) has risen to become the 5th commonest malignancy worldwide and the third leading cause of cancer-related death, exceeded only by cancers of the lung and stomach. Every year approximately 0.5–1 million new cases of HCC are diagnosed, causing 600 thousand deaths globally per year [1], [2]. China has one of the highest prevalent areas of HCC, mainly because of chronic hepatitis B carriers accounting for more than 10% of its population. However, only about 1/5 of hepatitis B virus (HBV) carriers are expected to develop HCC in their lifetime [3]. Therefore, host genetic factor may play important roles in hepatocarcinogenesis.

Glucose-regulated protein 78 (GRP78, 78 kDa), also called heat shock 70 kDa protein 5 (HSPA5), is a major endoplasmic reticulum (ER) chaperone and HSP70 family member that functions to bind and chaperone secretory proteins and promote dis-aggregation and proper protein folding and assembly [4], [5]. GRP78 is involved in the progression of HCC, and elevated GRP78 levels in tissues have been known to be related with poor prognosis [6]. Though *GRP78*, including single-nucleotide polymorphisms (SNPs) in its 3′ untranslated region (UTR), were not associated with HBV infection, our previous study also showed that a common SNP (rs430397 G>A) in the intron 5 of *GRP78* gene was associated with risk and prognosis of primary HCC [7], [8]. Especially, our recent study showed that the haplotypic block in its 3′ UTR (including rs16927997, rs1140763 and rs12009) were not associated with HCC risk [9]. We wonder whether these 3′ UTR variants are the contributing factors to HCC prognosis. Therefore, we investigated the associations of SNPs in the 3′ UTR of *GRP78* with overall survival among a Han Chinese population with HCC.

## Materials and Methods

### Patients

All participants provided written informed consents (from their guardians where necessary). This study was conducted in accordance with the tenets of the Declaration of Helsinki and its amendments and approved by the ethics committee of Guangzhou Medical College. The study population consisted of 576 HCC cases who inhabited in Guangzhou City or its neighboring townships at Guangdong province, a well-known high-risk region for HCC located in southern China between 1996 to 2003, which described previously [8]. The diagnosis of HCC was verified by liver histology, or based on the findings of radiological features suggestive of HCC in at least two image examinations including abdominal ultrasound, contrast enhanced dynamic computed tomography (CT), magnetic resonance imaging (MRI), and hepatic angiography, or by a single positive imaging technique associated with serum *α*-fetoprotein (AFP) level ≥25 µg/L. All patients were shown not to have other cancers by an initial screening examination.

### Genotyping

Genomic DNA was extracted from peripheral blood leukocytes using QIAGEN QIAamp DNA Mini Blood Kit (Hilden, Germany). Three validated SNPs (rs16927997 T>C, rs1140763 T>C and rs12009 T>C) in the 3′ UTR of GRP78 gene were detected by the TaqMan Assay-by-Design service (Applied Biosystems, Foster City, CA). The details of sequences and reaction conditions are available upon request (https://products.appliedbiosystems.com/ab/en/US/adirect/ab). PCR was performed using the TaqMan Universal Master Mix without UNG on the ABI PRISM 7900HT Sequence Detection System (Applied Biosystems, Foster City, CA) and heated to 95°C for 10 minutes followed by 40 cycles of 92°C for 15 seconds and 60°C for 1 minute, as described previously.

### Follow-up

The patients were followed up for a range of 6∼66 months. Data were collected from at least one of the following sources: the hospitals inpatient and outpatient records (staff periodically review computerized hospital tumor registry data or medical records for updated information), primary physician's offices, and patient or family contact. When it is observed that a patient has had no recent visit, the general practitioner of the patient is contacted to ascertain the patient's vital status. The primary endpoint was overall survival duration from the date of HCC diagnosis to the date of death. Patients who were alive at the end of the study period were censored on the last date of follow-up. The end of the observation period is the end of April 2008. In the period of analysis, 331 patients had died within 5 years of diagnosis, 35 patients were alive and 62 patients lost follow-up.

### Statistical analysis

The distribution of genotype was tested for Hardy-Weinberg equilibrium with the Pearson Chi-square test. Haplotypes were constructed by Haploview version 3.2 [10]. The diplotypes were constructed on the base of SNPs found in this study. Associations between haplotype distribution and clinicopathological variables were also assessed by the Chi-square test. The Kaplan-Meier method was used to estimate overall survival by allele, genotype, haplotype or diplotype and the log-rank test was used to compare the survival distributions. The wild-type allele/genotype and the corresponding haplotype/diplotype were taken as the references. To account for multiple testing, the Bonferroni correction was applied. All statistical evaluations were done using SPSS version 13.0 (SPSS Inc, Chicago, IL, USA). All tests were two-tailed, and *P* or *P*_corrected_ <0.05 was considered statistically significant.

## Results

Baseline characteristics for the three haplotype groups are presented in Table 1. Among the 576 patients in this study, 344 (59.7%) are male, and 490 (85.1%) are cirrhosis. With tumor, nodes, metastasis-classification (TNM) stage, 173 (30.0%) had advanced stage (III/IV) and 403 (70.0%) of the patients had primary stage (I/II). The genotype frequencies were in good agreement with the expected genotype distributions and consistent with Hardy-Weinberg equilibrium in cases (data not showed). By grouping the three SNPs together, three haplotypes with frequency larger than 1% were found in case population according linkage disequilibrium (LD) pattern. LD analysis further revealed that all selected SNPs were in strong LD with pairwise D' = 1, and formed a haplotype block according to the internally developed solid spine of LD in our study population. The analyzed haplotype distribution was as follows: 614 (53.3%) T-T-T, 461 (40.0%) T-C-C, and 77 (6.7%) C-C-T. The distribution of haplotype was not related to age, gender, HBsAg, AFP, tumor size, cirrhosis, or TNM stage (*P*>0.05, respectively).

**Table 1:** Demographic characteristics and haplotypic distributions of the 3′ untranslated region (UTR) of *GRP78* gene among cases with HCC.

Characteristics		T-T-T		T-C-C		C-C-T		*p* *
		No.	%	No.	%	No.	%	
Age, years	≤40	83	13.5	55	11.9	8	10.4	0.872
	>40, ≤60	309	50.3	230	49.9	39	50.6	
	>60	222	36.2	176	38.2	30	39.0	
Sex	Female	261	42.5	178	38.6	25	32.5	0.153
	Male	353	57.5	283	61.4	52	67.5	
HBV	HBsAg (−)	175	28.5	113	24.5	18	23.4	0.276
	HBsAg (+)	439	71.5	348	75.5	59	76.6	
Serum AFP	<25 ng/ml	97	15.8	83	18.0	6	7.8	0.074
	≥25 ng/ml	517	84.2	378	82.0	71	92.2	
Tumor size (cm)	≤5	135	22.0	111	24.1	20	26.0	0.874
	>5, ≤10	340	55.4	248	53.8	42	54.5	
	>10	139	22.6	102	22.1	15	19.5	
Cirrhosis	No	102	16.6	64	13.9	6	7.8	0.088
	Yes	512	83.4	397	86.1	71	92.2	
TNM stage	I	175	28.5	128	27.8	19	24.7	0.310
	II	271	44.1	178	38.6	35	45.4	
	III	110	17.9	105	22.8	13	16.9	
	IV	58	9.4	50	10.8	10	13.0	

*Chi-square test.

AFP, *α*-fetoprotein; HBV, hepatitis B virus; HCC, hepatocellular carcinoma; TNM, tumor, nodes, metastasis-classification.

**Figure 1:**
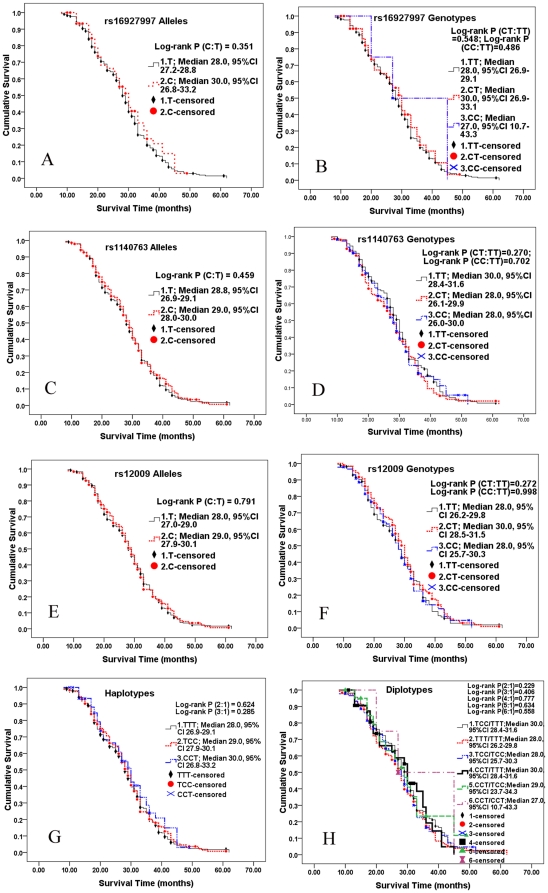
Kaplan-Meier survival curves show that overall survivals of 576 cases with HCC were not associated with the different alleles, genotypes, haplotypes and diplotypes in the *GRP78* gene including rs16927997, rs1140763 and rs12009. *P* value was calculated using a log-rank test. The wildtype alleles, homozygotes, and the corresponding haplotype and diplotype were designated as the referent.

The overall 5-year survival rate of the 576 patients was 10.4%. Univariate analysis of prognostic significance of the polymorphisms was performed for overall survival by the Kaplan-Meier method. We did not find any overall survival difference between the wildtype allele/genotype and mutant allele/genotype in rs16927997, rs1140763 and rs12009 (Log-rank *P*>0.05, respectively, Fig.1 A∼F). Conformed to the results of prognosis in allele and genotype levels, there were no significant differences in overall survival comparing the other haplotypes or diplotypes with the referred haplotype TTT or diplotype TCC/TTT (Log-rank *P*>0.05, respectively, Fig. 1 G or H).

The patients were divided into two subgroups according to the clinical characteristics or cutoff values of serum AFP. Survival curves were compared between the two subgroups. Table 2 lists the prognostic factors of the patients and shows the results of the univariate survival analysis. HBV infection and cirrhosis were found to be possible prognostic factors for patients with HCC (Log-rank *P* = 0.033 and 0.018, respectively). After Bonferroni correction in multiple comparisons, none of the clinical features showed a significant association (*P*_corrected_>0.05) with overall patient outcome. However, HBV infection status and cirrhosis showed the strongest association with survival.

**Table 2:** Univariate survival analyses of possible prognostic factors.

Subgroups		N	Univariate		
			5-years survival rate	Log-rank *P*	*P*_corrected_
Age	≤50 (years)	217	13.6%		
	>50 (years)	359	9.4%	NS	NS
Gender	Females	232	12.0%		
	Males	344	9.1%	NS	NS
HBV infection	HBsAg (−)	153	14.5%		
	HBsAg (+)	423	8.6%	0.033	NS
Serum AFP	Low (<25 ng/ml)	93	13.4%		
	High (≥25 ng/ml)	483	8.9%	NS	NS
Tumor size	≤5 (cm)	133	14.0%		
	>5 (cm)	443	9.0%	NS	NS
Cirrhosis	No	86	15.2%		
	Yes	490	8.7%	0.018	NS
TNM stage	I/II	403	13.6%		
	III/IV	173	8.4%	NS	NS

AFP, *α*-fetoprotein; HBV, hepatitis B virus; NS, not significant; TNM, tumor, nodes, metastasis-classification.

## Discussion

HCC is the most common primary epithelial malignancy occurring in the liver. It is a malignant tumor that arises from hepatocytes and is characterized by a trabecular, plate-like, or sinusoidal growth patterns with vascular invasion. In microscopic morphology, the tumor cells of HCC exhibit varying degrees of hepatocellular differentiation [11], [12]. Though remarkable improvements have been done in last decade on the understanding the molecular mechanisms involved in liver oncogenesis, the prognosis of patients affected by HCC is still poor for most of them [13].

In this study, we demonstrated for the first time the prognostic role of *GRP78* 3′ UTR polymorphisms in patients with resectable HCC. The attribution of variant haplotype was not related to demographic characteristics. Univariate analysis showed that the age, gender, tumor size, TNM stage, serum AFP, HBV, cirrhosis and the 3′ UTR polymorphisms (allele, genotype, haplotype and diplotype) are not independent prognostic factors for HCC in this population. Our hypothesis, based on the result displaying an involvement of these genetic variants in the susceptibility to HCC, was tested on a large cohort of prospectively followed-up patients with a large number of events allowing us to be confident in such a conclusion.

Large number of data showed that allele variants in certain genes are diagnostic and/or prognostic markers for primary HCC [14], [15], [16], [17], [18]. And the genetic difference of the gene related to disease process or survival may help us to predict each individual's susceptibility of developing serious disease and/or predict prognosis that can improve the cure rate substantially [19], [20], [21]. Therefore, there is intense interest in gaining a better understanding of the hepatocarcinogenesis to develop more reliable biomarkers to predict poor outcome. However, the genetic mechanisms for the progression of HCC remain largely unclear and need to be further established.

GRP78 is a central regulator of endoplasmic reticulum homeostasis due to its multiple functional roles, involving in polypeptide translocation across the ER membranes and acts as an apoptotic regulator by protecting the host cell against ER stress-induced cell death [22], [23]. Unfortunately, in many of the excellent studies, the exact mechanism is not known. However, we believe it is not a misleading statement that most of the cytoprotective effects of GRP78 come from the inhibition of stress-induced apoptosis [24].

As a hospital-based study, our patients with HCC were represent an unconsecutive series and were randomly selected, and the tumors all had been cured by surgical resect. And therefore inherent selection bias cannot be completely excluded. Overall however, our findings would suggest that there is no noteworthy influence of 3′ UTR variants in the *GRP78* on prognosis of resectable HCC in the Chinese Han population, despite the fact that the gene is considered an important participant in promoting tumor proliferation, survival, metastasis, and resistance to a wide variety of therapies. Our findings, however, require further verification from a much larger sample size with follow-up information on patient's survival outcomes.
